# Clinical features of serous retinopathy observed with cobimetinib in patients with *BRAF*-mutated melanoma treated in the randomized coBRIM study

**DOI:** 10.1186/s12967-017-1246-0

**Published:** 2017-06-24

**Authors:** Luis de la Cruz-Merino, Lorenza Di Guardo, Jean-Jacques Grob, Alfredo Venosa, James Larkin, Grant A. McArthur, Antoni Ribas, Paolo A. Ascierto, Jeffrey T. R. Evans, Antonio Gomez-Escobar, Giulio Barteselli, Susan Eng, Jessie J. Hsu, Anne Uyei, Brigitte Dréno

**Affiliations:** 1 Servicio de Oncología Médica, Hospital Universitario Virgen Macarena, Avenida Doctor Fedriani 3, 41071 Seville, Spain; 2 Fondazione IRCCS Istituto Nazionale dei Tumori, Milan, Italy; 3 Aix-Marseille University, Hôpital de la Timone, Marseille, France; 4 Ospedale Monaldi, Naples, Italy; 5 Royal Marsden NHS Foundation Trust, London, UK; 6 Peter MacCallum Cancer Centre, East Melbourne, VIC Australia; 7 University of Melbourne, Parkville, VIC Australia; 8 Jonsson Comprehensive Cancer Center at the University of California, Los Angeles, CA USA; 9 Istituto Nazionale Tumori Fondazione G. Pascale, Naples, Italy; 10 Beatson West of Scotland Cancer Centre, University of Glasgow, Glasgow, UK; 11 Genentech, Inc., South San Francisco, CA USA; 12 Nantes University, Nantes, France

**Keywords:** Cobimetinib, MEK inhibition, Melanoma, Serous retinopathy, Visual disturbance

## Abstract

**Background:**

Serous chorioretinopathy has been associated with MEK inhibitors, including cobimetinib. We describe the clinical features of serous retinopathy observed with cobimetinib in patients with *BRAF* ^V600^-mutated melanoma treated in the Phase III coBRIM study.

**Methods:**

In the coBRIM study, 493 patients were treated in two randomly assigned treatment groups: cobimetinib and vemurafenib (n = 247) or vemurafenib (n = 246). All patients underwent prospective ophthalmic examinations at screening, at regular intervals during the study, and whenever ocular symptoms developed. Patients with serous retinopathy were identified in the study database using a group of relevant and synonymous adverse event terms.

**Results:**

Eighty-six serous retinopathy events were reported in 70 patients (79 events in 63 cobimetinib and vemurafenib-treated patients vs seven events in seven vemurafenib-treated patients). Most patients with serous retinopathy identified by ophthalmic examination had no symptoms or had mild symptoms, among them reduced visual acuity, blurred vision, dyschromatopsia, and photophobia. Serous retinopathy usually occurred early during cobimetinib and vemurafenib treatment; median time to onset was 1.0 month. Most events were managed by observation and continuation of cobimetinib without dose modification and resolved or were resolving by the data cutoff date (19 Sept 2014).

**Conclusions:**

Cobimetinib treatment was associated with serous retinopathy in patients with *BRAF* ^V600^-mutated melanoma. Retinopathy was generally asymptomatic or mild. Periodic ophthalmologic evaluations at regular intervals and at the manifestation of any visual disturbance are recommended to facilitate early detection and resolution of serous retinopathy while patients are taking cobimetinib.

*Trial Registration* Clinicaltrials.gov (NCT01689519). First received: September 18, 2012

**Electronic supplementary material:**

The online version of this article (doi:10.1186/s12967-017-1246-0) contains supplementary material, which is available to authorized users.

## Background

Aberrant activation of the mitogen-activated protein kinase (MAPK) pathway is commonly observed in human cancers [1]. Approximately 50% of cutaneous melanomas harbor mutations in the *BRAF* gene, resulting in constitutive activation of the MAPK pathway [2, 3]. Combined BRAF and MEK inhibition enhances antitumor activity and may prevent or delay development of acquired resistance by providing more potent inhibition of the MAPK pathway [4, 5]. In the coBRIM study, the combination of cobimetinib, a MEK inhibitor, with vemurafenib, a BRAF inhibitor, significantly improved progression-free survival (PFS) [6] and overall survival (OS) [7] compared with placebo and vemurafenib (hereafter referred to as vemurafenib) in advanced *BRAF* ^V600^-mutated melanoma.

A unique ocular adverse event (AE) resembling central serous retinopathy has been described with MEK inhibitors [4, 8–13]. MEK-associated serous retinopathy manifests bilaterally within days after inhibitor initiation [9, 12, 14]. Patients may present with blurred or impaired vision, but many have no symptoms, with problems detected on ophthalmologic examination [9, 11]. Funduscopic examination reveals a blunted foveal light reflex and often multiple foveal lesions [9]. Optical coherence tomography (OCT) is used to detect bilateral subfoveal neurosensory retinal detachments not associated with fluorescein angiography or indocyanine green angiography, indicating unremarkable chorioretinal vasculature [9, 12].

In contrast, classic central serous retinopathy commonly manifests as unilateral metamorphopsia in middle-aged men, frequently associated with recent corticosteroid use or psychological stress [12, 15]. On funduscopic examination, typical findings include round, well-delineated, shallow, serous macular neurosensory detachment, often surrounded by a halo light reflex. Vascular abnormalities are usually found on fluorescein and indocyanine green angiography, exhibiting single or multiple discrete leakage points that evenly distribute dye throughout the subretinal fluid [15]. OCT reveals neurosensory detachment in addition to any associated retinal pigment epithelium detachment. The condition is typically self-limiting, and recovery of visual acuity is usually observed within 1–4 months without the need for treatment [15].

It is clear that the pathophysiology of MEK inhibitor-associated serous retinopathy is distinct from that of classic central serous retinopathy. Therefore, it is important to further define this AE. This article describes the clinical features of serous retinopathy observed with cobimetinib in patients treated in the coBRIM study.

## Methods

### Study design and treatment

coBRIM was a multicenter, randomized, double-blind, parallel, placebo-controlled Phase III study designed to evaluate the safety and efficacy of cobimetinib combined with vemurafenib, compared with vemurafenib, in patients with *BRAF* ^V600^ mutation—positive unresectable locally advanced or metastatic melanoma. This trial was registered on *clinicaltrials.gov* as NCT01689519. The primary end point was investigator-assessed PFS. Secondary end points included OS, objective response rate, duration of response, PFS as assessed by independent review, safety, pharmacokinetics, and health-related quality of life (HRQOL). Complete methodology of the study and primary efficacy and safety results have previously been published and the protocol is available online [6]. The study was approved by the institutional review board or ethics committee at each participating institution and was conducted in accordance with the provisions of the Declaration of Helsinki and the International Conference on Harmonisation guidelines for Good Clinical Practice. All the patients provided written informed consent.

Key eligibility criteria were age ≥18 years, histologically confirmed unresectable locally advanced stage IIIC or IV melanoma, *BRAF* ^V600^ mutation detected using the **cobas** ^**®**^ 4800 BRAF V600 Mutation Test (Roche Molecular Systems Inc. USA), no history of systemic therapy for advanced disease, measurable disease according to Response Evaluation Criteria In Solid Tumors version 1.1, and Eastern Cooperative Oncology Group performance status 0–1. Because of the known ocular toxicities associated with MEK inhibitors, patients with a history or ophthalmic examination evidence of a retinal abnormality considered a risk factor for neurosensory retinal detachment/central serous retinopathy, retinal vein occlusion, or neovascular macular degeneration were excluded. Patients also were excluded if they had risk factors for retinal vein occlusion, including uncontrolled glaucoma with intraocular pressure >21 mmHg, grade ≥2 serum cholesterol, hypertriglyceridemia, or fasting hyperglycemia.

Patients were randomly assigned in a 1:1 ratio using an interactive response system [Perceptive Informatics (now Parexel International), USA] to receive oral vemurafenib (960 mg twice daily) in combination with either placebo or oral cobimetinib (60 mg once daily for 21 days followed by 7 days off). Treatment was administered in 28-day cycles and continued until disease progression, unacceptable toxicity, or withdrawal of consent. Patients were stratified according to the American Joint Committee on Cancer stage and geographic region. Dose modifications for management of specific adverse events were mandated by the protocol. For grade ≥2 visual symptoms, a complete ophthalmic examination was to be performed and treatment with cobimetinib combined with vemurafenib was to be interrupted until resolution to grade ≤1. Dose reduction of the implicated agent was employed if grade ≥2 visual symptoms recurred. Treatment was to be permanently discontinued in the case of retinal vein occlusion, lack of resolution of visual symptoms to grade ≤1 within 28 days, or recurrence of grade ≥2 visual symptoms despite dose reduction.

### Ophthalmic assessments

Complete ophthalmic examinations were performed by a qualified ophthalmologist on all patients at screening and day 1 of cycle 2, then every three cycles until cycle 11, every four cycles until cycle 23, every six cycles thereafter or when clinically indicated during the study, and at the end of study treatment visit (Additional file 1). Examinations included visual acuity testing, intraocular pressure measurements by tonometry, slit-lamp ophthalmoscopy, indirect ophthalmoscopy, and OCT (time or spectral-domain).

### Analysis

To ensure collection of all potential events, patients who experienced serous retinopathy were identified using a broad group of preferred terms from the Medical Dictionary for Regulatory Activities (MedDRA) (Additional file 2) and record review from ophthalmologic examinations. Events were graded according to the National Cancer Institute Common Terminology Criteria for Adverse Events (NCI CTCAE) version 4.0 scale for eye disorders-other (Additional file 3). NCI CTCAE version 4.0 does not have a severity grading scale for serous retinopathy. Results were presented and tabulated with descriptive statistics. The data cutoff date for this analysis was 19 Sept 2014.

## Results

### Patients

Between January 2013 and January 2014, coBRIM enrolled 495 patients from 135 sites in the United States, Australia, New Zealand, Israel, and Europe. Of 495 randomly assigned patients, one patient in each arm did not receive study treatment (Additional file 4); the remaining 493 received cobimetinib combined with vemurafenib (n = 247) or vemurafenib (n = 246). At the time of data cutoff, the median follow-up duration for all patients was 10.7 months.

### Frequency and severity of serous retinopathy events

**Fig. 1:**
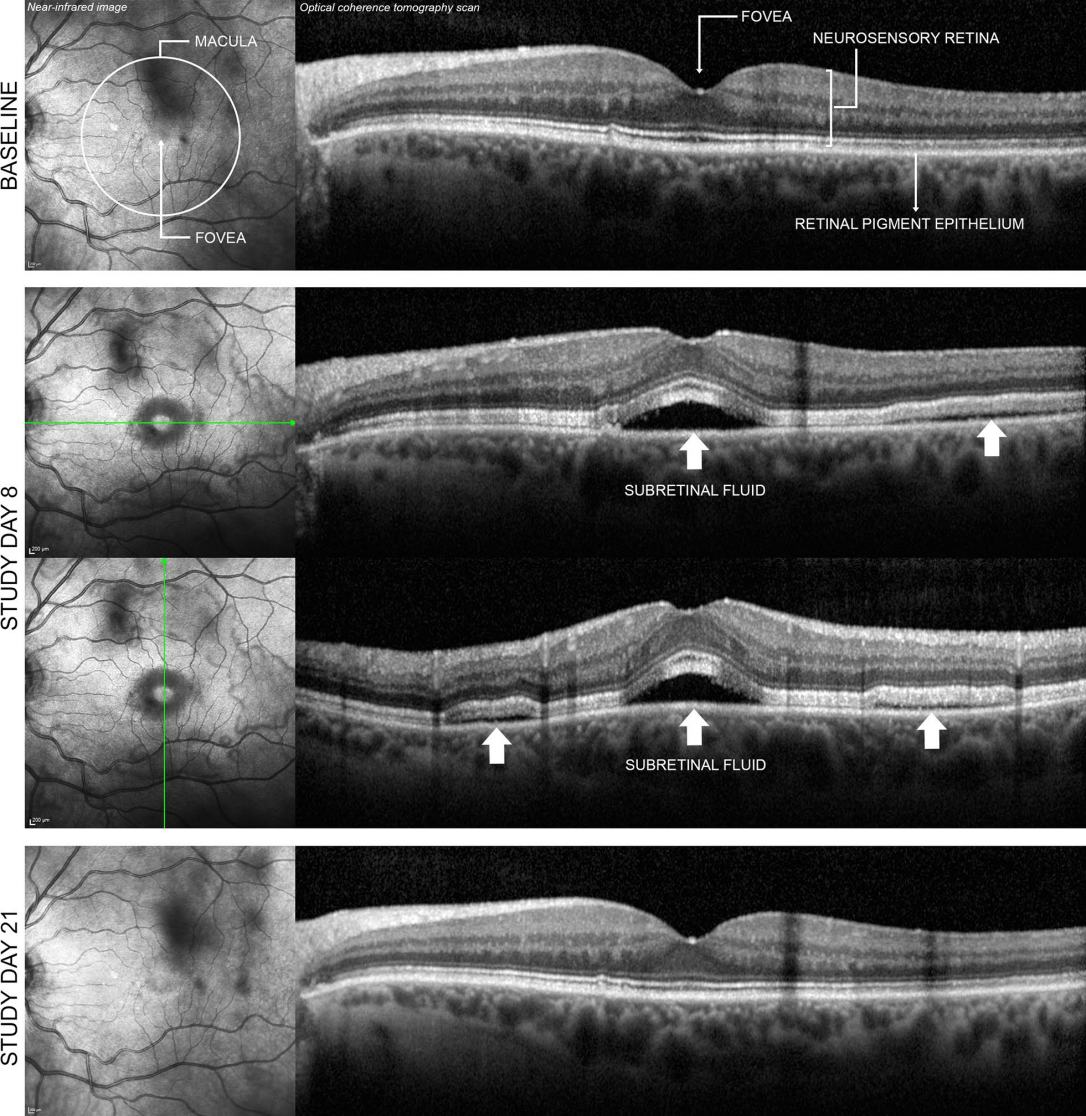
Bilateral serous retinopathy (*left eye* shown) in a 64-year-old woman receiving cobimetinib combined with vemurafenib. At baseline, ophthalmic examination findings were normal, and no retinal abnormalities were detected either on bidimensional near-infrared image of the macula or on cross-sectional optical coherence tomography scan across the fovea *(first row)*. On study day 8, the patient had nonserious grade 2 bilateral blurred vision. On study day 10, ocular examination with indirect ophthalmoscopy revealed bilateral serous subfoveal neurosensory detachment (i.e. subretinal fluid). Retinal imaging showed that detachments of the neurosensory retina were not limited to the fovea but were multiple and extended across the macular area *(second and third rows, white arrows)*. A diagnosis of nonserious grade 2 serous retinopathy was made, and no treatment was administered for this event. On study day 14, the event of neurosensory detachments was considered resolved on indirect ophthalmoscopy, and blurred vision was considered resolved on study day 15. On study day 21, retinal imaging confirmed resolution of the retinal abnormalities *(fourth row)*. The investigator considered blurred vision and serous retinopathy to be related to cobimetinib combined with vemurafenib. Treatment with cobimetinib combined with vemurafenib was permanently discontinued on study day 36 because of other adverse events, including pyrexia, rash, and elevated liver enzyme levels, with consideration of the previous event of serous retinopathy

Serous retinopathy occurred in 63 of 247 cobimetinib combined with vemurafenib recipients (26%) (79 events), compared with seven of 246 vemurafenib recipients (3%) (7 events). In the cobimetinib combined with vemurafenib arm, these events were usually reported as chorioretinopathy (49%) and retinal detachment (33%) (Table 1). Serous retinopathy events were characterized by accumulation of subretinal fluid (Fig. 1). Most patients had no symptoms or had mild symptoms (grade 1; see Table 1). Most events were bilateral and were identified by surveillance ophthalmic examination. Grade 3–4 serous retinopathy (Table 1) was identified in seven cobimetinib combined with vemurafenib recipients (11%); symptoms were present in all seven patients and included reduced visual acuity, blurred vision, dyschromatopsia, and photophobia.

**Table 1:** Frequency of adverse events by MedDRA preferred term

	Cobimetinib + vemurafenib (n = 247)	Placebo + vemurafenib (n = 246)
Total patients with serous retinopathy, n (%)	63 (25.5)	7 (2.8)
Total events, n	79	7
AE preferred term, n/N (%)		
Chorioretinopathy	31/63 (49.2)	1/7 (14.3)
Retinal detachment	21/63 (33.3)	1/7 (14.3)
Retinal pigment epithelium detachment	8/63 (12.7)	1/7 (14.3)
Macular edema	5/63 (7.9)	1/7 (14.3)
Macular fibrosis	2/63 (3.2)	1/7 (14.3)
Retinal disorder	1/63 (1.6)	2/7 (28.6)
Retinopathy	2/63 (3.2)	0
Macular retinal pigment epithelium detachment	1/63 (1.6)	0
NCI CTCAE grade, n/N (%)^a^		
Grade 1	33/63 (52.4)	6/7 (85.7)
Grade 2	23/63 (36.5)	1/7 (14.3)
Grade 3	6/63 (9.5)	0
Grade 4	1/63 (1.6)	0

*MedDRA* Medical Dictionary for Regulatory Activities, *AE* adverse event, *NCI CTCAE* National Cancer Institute Common Terminology Criteria for Adverse Events

^a^Highest severity grade per patient

Baseline characteristics of patients with serous retinopathy in the cobimetinib combined with vemurafenib arm were similar to those of the overall coBRIM population (Table 2). The frequency with which serous retinopathy developed in men and women in the cobimetinib combined with vemurafenib arm was 59% and 41%, respectively; classic serous retinopathy occurs predominantly in men [15].

**Table 2:** Baseline characteristics of patients with serous retinopathy compared with the overall coBRIM population

	Cobimetinib + vemurafenib recipients with SR (n = 63)	Placebo + vemurafenib recipients with SR (n = 7)	Overall coBRIM population (N = 493)
Age, years			
Median	59	62	55
Range	30–78	33–76	23–88
Sex, n (%)			
Male	37 (58.7)	2 (28.6)	286 (57.8)
Female	26 (41.3)	5 (71.4)	209 (42.2)
Region, n (%)			
Europe	51 (80.9)	5 (71.4)	366 (73.9)
Australia/New Zealand	8 (12.7)	2 (28.6)	78 (15.8)
North America	4 (6.4)	0	51 (10.3)

*SR* serous retinopathy

### Time to first onset of serous retinopathy

**Fig. 2:**
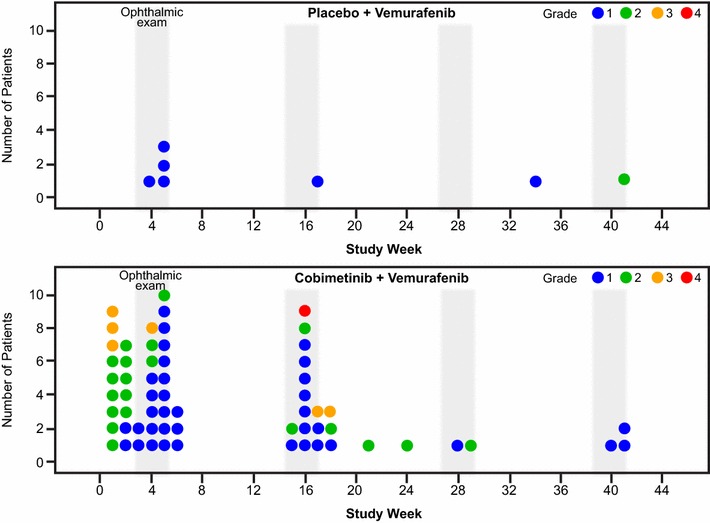
First onset and severity grade of serous retinopathy in the coBRIM study, per patient

Median time to first onset of serous retinopathy in the cobimetinib combined with vemurafenib arm was 1.0 month (range, 0.1–9.3 months). Most (63%) grade ≥2 events occurred during cycle 1 (Fig. 2).

### Management and resolution of serous retinopathy

Most patients with a first serous retinopathy event of grade 1 continued to receive cobimetinib without dose reduction (72%); it resolved in 38% of these patients (Table 3). In patients with grade 2 serous retinopathy (65%), doses were frequently reduced; it resolved in 92% of these patients. Grade 3–4 serous retinopathy was managed by interruption or withdrawal of cobimetinib and was considered resolved or resolving at data cutoff; only one patient needed surgical treatment. This patient had grade 3 idiopathic rhegmatogenous retinal detachment of the right eye considered by the investigator to be unrelated to cobimetinib or vemurafenib. Although clinically distinct from serous retinopathy, it was included with the cases of serous retinopathy because retinal detachment was included in preselected MedDRA preferred terms relevant to serous retinopathy (Additional file 2). This patient underwent pneumatic retinopexy, a surgical treatment for localized rhegmatogenous retinal detachment, and the event was considered resolved on the day of surgery.

**Table 3:** Cobimetinib dose modification after the first event of serous retinopathy in the cobimetinib plus vemurafenib arm

Action taken with cobimetinib	All patients with serous retinopathy events (n = 63)		Patients with grade 1 events (n = 36)		Patients with grade 2 events (n = 20)		Patients with grade 3 events (n = 6)		Patients with grade 4 events (n = 1)	
	All	Resolved	All	Resolved	All	Resolved	All	Resolved	All	Resolved
Dose not changed, n	30	12	26	10	3	1	1	1	0	0
Dose reduced, n	18	16	4	3	13	12	1	1	0	0
Drug interrupted, n	10	5	5	3	2	0	3	2	0	0
Drug withdrawn, n	4	1	0	0	2	1	1^a^	0	1^a^	0
Unknown, n	1	0	1	0	0	0	0	0	0	0

^a^Patients had events that were considered “recovering/resolving”

Overall, 52% of serous retinopathy events in the cobimetinib combined with vemurafenib arm had resolved at data cutoff. Of the resolved events, median time to resolution was 1.2 months (range, 0.2–10.3 months).

### Recurrent serous retinopathy

Fourteen patients with recurrent serous retinopathy were in the cobimetinib and vemurafenib arm. One patient experienced three recurrent episodes; the remaining 13 experienced one recurrence each.

Most subsequent events were of the same or milder grade than the initial event; however, in three patients, initial and recurrent events were grade 1 and 2, respectively. No recurrent events were grade ≥3. At the data cutoff date, half the recurrent events had resolved. No pattern emerged of action taken for recurrent events; patients continued taking cobimetinib combined with vemurafenib, therapy was interrupted, or therapy was withdrawn (four patients).

## Discussion

This article describes clinical characteristics of serous retinopathy in patients treated with cobimetinib combined with vemurafenib in the coBRIM study. Because all patients were prospectively screened for visual disturbances throughout the study, per protocol, most events were diagnosed early in the course of treatment, and most patients had no or mild symptoms at diagnosis.

The clinical relevance of surveillance ophthalmic examinations is unclear. Most patients with serous retinopathy had no symptoms, did not require drug discontinuation or dose alteration, and did not experience greater severity of the condition over time. Patients with mildly symptomatic (grade 2) serous retinopathy had dose reduction, resulting in resolution in the majority. For all but one patient (rhegmatogenous retinal detachment) with grade ≥3 serous retinopathy, dose interruption or withdrawal led to improvement without surgery. Few patients experienced recurrence despite continuing or restarting cobimetinib. All recurrent serous retinopathy events were either asymptomatic or mildly symptomatic, and half resolved by the time of data cutoff.

Based on the limited scientific literature available, characteristics of serous retinopathy observed with cobimetinib were similar to those of retinal changes observed with other MEK inhibitors [9, 11, 12, 14], suggesting that this is a class effect [11]. Symptomatic ocular toxicity, most commonly blurred vision, has been reported in 0–17% of patients treated with single-agent MEK inhibitors [16], whereas blurred vision and chorioretinopathy were reported in 0–2% and 1% of patients, respectively, treated with the combination of trametinib and dabrafenib [17, 18]. Higher incidences of ocular toxicity have been observed in trials incorporating prospective screening, but the reported events are frequently asymptomatic. Prospective screening identified retinopathy in 59% of patients treated with binimetinib, alone or in combination with RAF265 or encorafenib, with symptomatic events reported in 25% of patients [9], and 26% of patients treated with cobimetinib combined with vemurafenib in the current study, with symptomatic (grade ≥ 2) events in 12% of patients. Given the lack of prospective screening for retinopathy in other trials, incidence cannot be compared across MEK inhibitors. Because of a lack of randomized trials and differences in the description and reporting of ocular toxicity across trials, it is unclear whether the combination of a BRAF inhibitor with a MEK inhibitor alters the incidence of ocular toxicities compared with MEK inhibitor monotherapy. However, in one small study, addition of the pan-RAF inhibitor RAF265 or the selective BRAF inhibitor encorafenib to the MEK inhibitor binimetinib did not appear to influence the incidence of retinopathy [9]. Furthermore, anecdotal data with binimetinib suggest a temporal relationship between ocular toxicity and dose administration/blood binimetinib levels [9]. Further research is necessary to determine whether a similar relationship exists for other MEK inhibitors.

Although the MAPK pathway seems to play an important role in maintenance, protection, and repair of the retina, pathophysiologic mechanisms underlying MEK inhibitor-induced retinopathy are not well understood [11]. The retinal pigment epithelium forms the outer component of the blood–retina barrier and controls solute and fluid permeability, a function critical for maintenance of the specialized environment of the neural retina [19]; fluid accumulation between these layers can result in retinal detachment [11]. There is evidence that MAPK signaling regulates density of fluid transport channels (aquaporins) between retinal pigment epithelial cells [20]. Thus, MEK inhibition may alter permeability of the retinal pigment epithelium, allowing accumulation of subretinal fluid. Further study is needed to test this hypothesis.

In other retinal diseases, the long-term presence of subretinal fluid can lead to photoreceptor death and permanent vision loss; therefore, persistent events may warrant treatment [15]. Understanding of the pathogenesis of classic central serous chorioretinopathy emphasizes the role of the choroid, which seems to be extremely permeable in this disease [15]. However, chorioretinal abnormalities are not observed in MEK inhibitor-associated serous retinopathy, in which pathophysiology seems to result instead from altered permeability of the retinal pigment epithelium. Therefore, treatments that might promote subretinal fluid resolution in classic central serous chorioretinopathy [15] may not be useful for subretinal fluid resolution in MEK inhibitor-associated serous retinopathy. It is reassuring that almost all cases of MEK inhibitor-associated serous retinopathy in the coBRIM study ultimately resolved either spontaneously or through dose modification.

In our experience, management of cobimetinib-associated serous retinopathy necessitates close collaboration with an ophthalmologist, and early involvement should be considered because most events occur within 1 month of treatment initiation. However, prospective screening is probably not warranted because surveillance ophthalmologic examination typically identifies asymptomatic serous retinopathy. Ophthalmologic examination should be performed periodically during cobimetinib treatment and as necessary for new or worsening visual disturbances. Temporarily withholding or reducing the dose of cobimetinib should be considered upon diagnosis of serous retinopathy.

There are several limitations to our results. First, reliance on AE reporting rather than ophthalmic (OCT) confirmation might have resulted in underreporting of asymptomatic or mildly symptomatic cases. Second, ophthalmic images were not prospectively centrally reviewed. Third, visual acuity was not measured in a standardized fashion across sites. Despite these limitations, the large difference in rates of serous retinopathy between the cobimetinib combined with vemurafenib arm and the vemurafenib arm make it unlikely that results were caused by study limitations rather than the real effects of MEK inhibition with cobimetinib.

## Conclusion

This report provides context around the guidance for the management of serous retinopathy in patients with unresectable or metastatic melanoma with a *BRAF* V600E or V600K mutation receiving cobimetinib in combination with vemurafenib. Cobimetinib in combination with vemurafenib was associated with serous retinopathy in 26% of patients with *BRAF* ^V600^-mutated melanoma. Although retinopathy was generally asymptomatic or mild and although most affected patients could safely continue to receive cobimetinib, dose interruption and dose reduction should be considered. Patients receiving cobimetinib should undergo ophthalmologic examination at regular intervals and whenever they experience symptoms suggestive of serous retinopathy. Additional examination may be warranted if symptoms persist. Guidance on the management of MEK-associated serous retinopathy can be found in the local prescribing information where cobimetinib is approved.

## Additional files

**Additional file 1.** Study drug dosing and ophthalmic examination schedule.

**Additional file 2.** MedDRA-preferred terms relevant to serous retinopathy.

**Additional file 3.** NCI CTCAE v4.0 scale for eye disorders–other.

**Additional file 4.** CONSORT diagram.

## Abbreviations

MAPK: mitogen-activated protein kinase
PFS: progression-free survival
OS: overall survival
AE: adverse event
OCT: optical coherence tomography
HRQOL: health-related quality of life
MedDRA: Medical Dictionary for Regulatory Activities
NCI CTCAE: National Cancer Institute Common Terminology for Adverse Events
