# Supplementary figures and images for: Clinical features of serous retinopathy observed with cobimetinib in patients with BRAF-mutated melanoma treated in the randomized coBRIM study
Source: J Transl Med. 2017 Jun 24;15:146. doi: 10.1186/s12967-017-1246-0 (PMC5483259; doi:10.1186/s12967-017-1246-0)

**Additional file 1.** coBRIM study drug dosing and ophthalmic examination schedule.


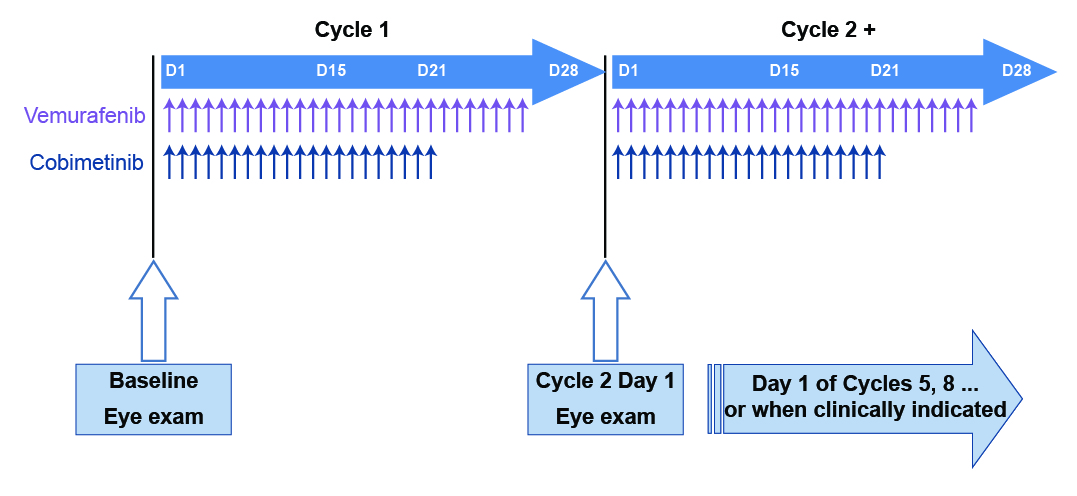

Supplement: Supplementary file 1 — Additional file 1. Study drug dosing and ophthalmic examination schedule. [file 12967_2017_1246_MOESM1_ESM.docx]

**Additional file 4** CONSORT diagram (data cutoff date 19 Sept 2014)

##
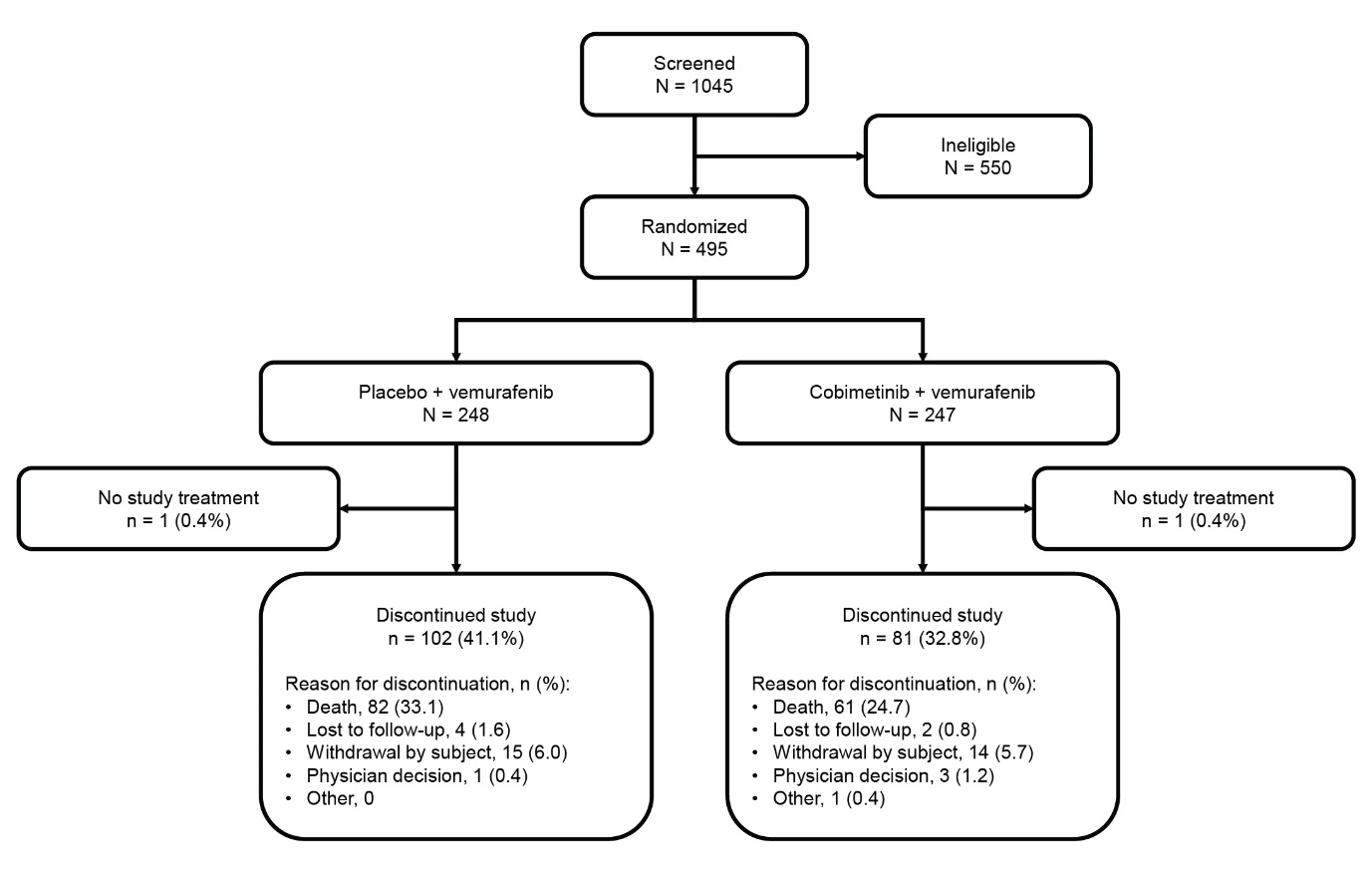

Supplement: Supplementary file 4 — Additional file 4. CONSORT diagram. [file 12967_2017_1246_MOESM4_ESM.docx]
