# Supplementary material for: Clinical features of serous retinopathy observed with cobimetinib in patients with BRAF-mutated melanoma treated in the randomized coBRIM study
Source: J Transl Med. 2017 Jun 24;15:146. doi: 10.1186/s12967-017-1246-0 (PMC5483259; doi:10.1186/s12967-017-1246-0)
Supplement: Supplementary file 2 — Additional file 2. MedDRA-preferred terms relevant to serous retinopathy. [file 12967_2017_1246_MOESM2_ESM.docx]

**Additional file 2** MedDRA-preferred terms relevant to serous retinopathy

| Chorioretinal disorder |
| --- |
| Chorioretinopathy |
| Detachment of macular retinal pigment epithelium |
| Detachment of retinal pigment epithelium |
| Macular fibrosis |
| Macular edema |
| Retinal detachment |
| Retinal disorder |
| Retinal pigment epithelium tear |
| Retinal tear |
| Retinopathy |
| Scleral buckling surgery |

*MedDRA* Medical Dictionary for Regulatory Activities
