# Supplementary material for: Clinical features of serous retinopathy observed with cobimetinib in patients with BRAF-mutated melanoma treated in the randomized coBRIM study
Source: J Transl Med. 2017 Jun 24;15:146. doi: 10.1186/s12967-017-1246-0 (PMC5483259; doi:10.1186/s12967-017-1246-0)
Supplement: Supplementary file 3 — Additional file 3. NCI CTCAE v4.0 scale for eye disorders–other. [file 12967_2017_1246_MOESM3_ESM.docx]

**Additional file 3** National Cancer Institute Common Terminology Criteria for Adverse Events version 4.0 scale for eye disorders–other

| **Grade** | **Symptoms** | **Intervention** |
| --- | --- | --- |
| 1 | Asymptomatic or mild; clinical or diagnostic observations only | Not indicated |
| 2 | Moderate; limiting age-appropriate instrumental activities of daily living | Minimal; local or noninvasive |
| 3 | Severe or medically significant but not immediately sight threatening; disabling; limiting self-care activities of daily living | Hospitalization or prolongation of existing hospitalization |
| 4 | Sight-threatening consequences; blindness (20/200 or worse) in the affected eye | Urgent and immediate |
